# A Middle-Aged Woman with Logopenic Progressive Aphasia as a Precursor of Alzheimer's Disease: Case Report and Review of the Literature

**DOI:** 10.1155/2011/450301

**Published:** 2011-09-29

**Authors:** Stephanie M. Awad, Amer M. Awad

**Affiliations:** ^1^Family Medicine Residency Program, Baton Rouge General Medical Center, Baton Rouge, LA 70806, USA; ^2^Baton Rouge Neurology Associates, Baton Rouge General Medical Center, 3600 Florida Boulevard, Baton Rouge, LA 70806, USA

## Abstract

Primary progressive aphasia is a neurodegenerative disorder that was recently classified into three types: fluent (semantic), nonfluent, and logopenic. The logopenic variant is the least common one and is closely related to Alzheimer's disease in comparison to the other two variants that are closely related to frontotemporal dementia. We report the case of a middle-aged woman who presented to our center with progressive aphasia that was undiagnosed for two years. The patient's neurological evaluation including positron emission tomography is consistent with a logopenic variant of primary progressive aphasia.

## 1. Introduction

Primary progressive aphasia (PPA) is a spectrum of heterogeneous disorders that are characterized by slowly progressive neurodegeneration affecting mainly the language function [1].

The first description of isolated language deteriorations was probably made by Serieux in the late nineteenth century [2]. Comprehensive epidemiological studies are so far lacking to define the exact demographics of PPA. However, PPA is still considered a rare disease with variable progression rates, variable ages of onset and without remarkable gender preponderance.

There has been dramatic progress in our understanding of PPA in the last few decades thanks to the progress in neuropathology, neurogenetics, and neuropsychology. PPA was recently subclassified into three distinct types [3, 4]: progressive nonfluent aphasia (PNFA), semantic dementia (SD), and the recently described logopenic variant (LPA). The logopenic variant accounts for one-third of PPA cases [3]. In this paper we will discuss the case of a patient with LPA that has early Alzheimer's disease features as well.

## 2. Case Report

The patient is a 54-year-old left-handed Caucasian lady who was referred to our center for evaluation of speech difficulties. The patient noted a gradually progressing speech problem about two years prior to her presentation. Her main difficulties were related to word finding and inability to express herself very well with frequent pauses. Her comprehension was also affected, but much less than her fluency. In addition, she noted that her reading abilities were declining and her writing skills seemed to be deteriorating. The patient denied any history of weakness, trouble swallowing, trouble breathing, numbness, loss of vision, hearing, or balance. Her husband had thought that her short-term memory was also impaired. The patient's family history was significant for Alzheimer's disease that affected her aunt in her 80s. The patient denied any history of strokes, seizures, or head injury. No behavioral abnormalities were reported. Her medical exam was normal, except for high cortical abnormalities. Her cranial nerves, motor system, sensory system, and coordination system exams were normal. The patient scored 23/30 on the Montreal Cognitive Assessment Exam (MOCA). There was evidence of aphasia on detailed language examination that can be classified as global aphasia. The patient's fluency was decreased with word finding difficulties without agrammatism. Comprehension of isolated words was intact, whereas comprehension of complex sentences was impaired. Repetition and digit span was impaired as well. Naming was mildly affected. Short-term memory including episodic memory was impaired. Cues did not seem to help improve recall. Interestingly, visuospatial function was impaired in a very subtle way. The patient was able to copy a cube but only after several unsuccessful attempts.

**Figure 1:**
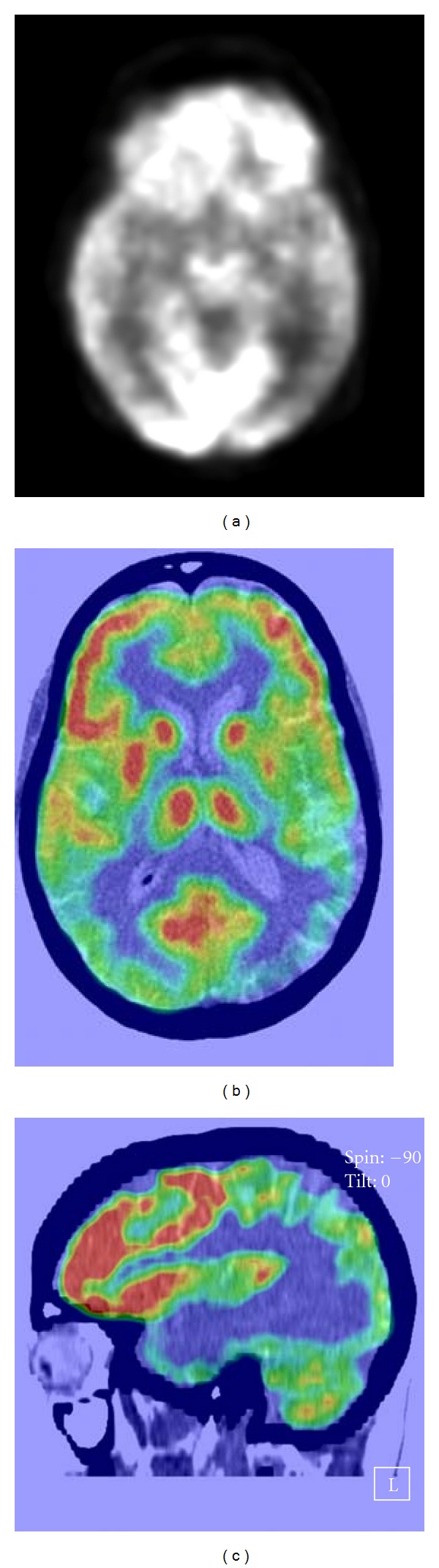
(a)–(c) show brain PET scan of the patient showing bilateral, predominantly left-sided, parietotemporal hypometabolism in different orientations.

Prior to being evaluated by us the patient underwent numerous tests that were reported to be normal including brain magnetic resonance imaging (MRI), comprehensive autoimmune panel, electroencephalography (EEG) and vitamin B12, folic acid, thyroid stimulating hormone (TSH), and rapid plasma reagin (RPR) tests. We evaluated the patient with positron emission tomography (PET) scan which showed hypometabolism in the bilateral parietal as well as temporal lobes (Figures 1(a), 1(b), and 1(c)).

 The clinical findings along with the radiological findings are highly suggestive of logopenic primary progressive aphasia (LPA).

## 3. Discussion

Our patient's neurocognitive assessment is highly suggestive of LPA.

LPA was first described by Gorno-Tempini and his coworkers in 2004 [3]. The disorder typically presents with word finding difficulty with no agrammatism, impaired repetition, impaired comprehension of complex sentences with retained comprehension of isolated words [3, 4].

LPA typically involves an abnormality in the parietotemporal lobes, predominantly, the dominant side [3–6]. Our patient's PET scan revealed bilateral parietotemporal pathology, predominantly in the left side. This was consistent with the neuroimaging findings.

The majority of LPA cases show AD cerebrospinal fluid (CSF) biomarkers [7, 8]. CSF biomarkers were not yet available at the time of case reporting.

The histopathology of LPA is variable, but pathology of Alzheimer's disease is the most common finding. In the series reported in 2008 by Josephs and coinvestigators, all PPA patients whose pathology showed changes consistent with AD pathology belonged to the LPA group [9]. Other series found AD pathology in the majority of cases, including frontotemporal lobar dementia (FTLD) pathology, in about one-fifth of LPA phenotype [7, 8, 10, 11].

Our patient had very subtle findings that suggest very early AD-like mild visuospatial dysfunction and subtle cortical memory deficits. These findings predict AD-type pathology.

## 4. Conclusion

LPA is a rare neurodegenerative disorder that is closely related to Alzheimer's disease. The early symptoms are very subtle and require a high index of suspicion. Healthcare providers need to be aware of this entity and other entities that present with subtle cognitive abnormalities. Despite the lack of effective treatment, recruiting these patients to research is invaluable to help improve our understanding of the pathophysiology of the disease that should guide us one day to an effective treatment.
